# Gut microbiota dysfunction in Crohn’s disease

**DOI:** 10.3389/fcimb.2025.1540352

**Published:** 2025-02-11

**Authors:** Sylvie Buffet-Bataillon, Gabriela Durão, Isabelle Le Huërou-Luron, Olivier Rué, Yann Le Cunff, Vincent Cattoir, Guillaume Bouguen

**Affiliations:** ^1^ Department of Clinical Microbiology, CHU Rennes, Rennes, France; ^2^ Institut NUMECAN, INRAE, INSERM, Univ Rennes, Rennes, France; ^3^ Université Paris-Saclay, INRAE, MaIAGE, Jouy-en-Josas, France; ^4^ Université Paris-Saclay, INRAE, BioinfOmics, MIGALE Bioinformatics Facility, Jouy-en-Josas, France; ^5^ IRISA, Univ Rennes, Inria, CNRS, Rennes, France; ^6^ CHU Rennes, Univ Rennes, INSERM, U1230, Rennes, France

**Keywords:** Crohn’s disease, microbiota, metabolic functions, treatment, oxidative stress

## Abstract

**Introduction:**

Crohn’s disease (CD) results from alterations in the gut microbiota and the immune system. However, the exact metabolic dysfunctions of the gut microbiota during CD are still unclear. Here, we investigated metagenomic functions using PICRUSt2 during the course of CD to better understand microbiota-related disease mechanisms and provide new insights for novel therapeutic strategies.

**Methods:**

We performed 16S rRNA-based microbial profiling of 567 faecal samples collected from a cohort of 383 CD patients, including 291 remissions (CR), 177 mild-moderate (CM) and 99 severe (CS) disease states. Gene and pathway composition was assessed using PICRUSt2 analyses of 16S data.

**Results:**

As expected, changes in alpha and beta diversity, in interaction networks and increases in Proteobacteria abundance were associated with disease severity. However, microbial function was more consistently disrupted than composition from CR, to CM and then to CS. Major shifts in oxidative stress pathways and reduced carbohydrate and amino acid metabolism in favour of nutrient transport were identified in CS compared to CR. Virulence factors involved in host invasion, host evasion and inflammation were also increased in CS.

**Conclusions:**

This functional metagenomic information provides new insights into community-wide microbial processes and pathways associated with CD pathogenesis. This study paves the way for new advanced strategies to rebalance gut microbiota and/or eliminate oxidative stress, and biofilm to downregulate gut inflammation.

## Introduction

Crohn’s disease (CD) is a chronic and relapsing gastrointestinal inflammatory disease triggered by both innate and adaptive immune responses to environmental factors in genetically-predisposed individuals (Manichanh et al., 2006; Dicksved et al., 2008). Although it remains unclear whether dysbiosis is a cause or a consequence of CD, it is thought to play a key role in the pathogenesis of CD (Ni et al., 2017). Numerous studies showed that the microbiota composition of faecal samples from CD patients was significantly different from that of non-CD controls (Joossens et al., 2011; Machiels et al., 2014; Vieira-Silva et al., 2019). This dysbiosis was characterised by low microbial alpha diversity (Pascal et al., 2017a; Deleu et al., 2021a), including reducing butyrate-producing bacteria identified as essential for gut homeostasis (Sokol et al., 2008). In the remission state, the microbiota usually turns back to eubiosis (Swidsinski et al., 2008; Papa et al., 2012; Gevers et al., 2014; Machiels et al., 2014; Kolho et al., 2015; Tedjo et al., 2016a; Franzosa et al., 2019). Furthermore, the higher likelihood of a favourable outcome of therapies is associated with an increase in short-chain fatty acid-producing bacteria and a decrease in mucus-decomposing bacteria (Kolho et al., 2015; Shaw et al., 2016; Ananthakrishnan et al., 2017; Doherty et al., 2018), as well as a decrease in bacteria with pro-inflammatory properties, such as *Fusobacterium*, *Escherichia*, *Veillonella*, *Streptococcus* (Shaw et al., 2016; Tedjo et al., 2016a; Zhou et al., 2018; Vieira-Silva et al., 2019).

Most treatments for CD target the immune response to reduce inflammatory responses (Lamb et al., 2019; Cushing and Higgins, 2021). Despite the use of several classes of advanced therapies, remission rates for all agents rarely exceed 30% (Kayal et al., 2023). This highlights both the large unmet medical need in CD for existing therapies and the need for new, complementary and highly effective medical therapies for CD.

In order to provide new insights for new treatments, the identification of the major perturbations in fundamental microbial metabolic functions is a prerequisite (Morgan et al., 2012). To address these challenges, gene marker approaches such as 16S rRNA are widely used to identify dysbiosis. To overcome the lack of functional information when using 16S rRNA profiling, tools such as Phylogenetic Investigation of Communities by Reconstruction of Unobserved States 2 (PICRUSt2) (https://github.com/picrust/picrust2) have been developed to predict the functional potential of a bacterial community (Langille et al., 2013; Douglas et al., 2020).

Therefore, the aim of this study was to characterise dysbiosis and identify microbial metabolic functions using PICRUSt2 in faecal samples from CD patients in remission, mild-moderate and severe disease, in order to provide new insights for novel therapeutic strategies targeting and/or involving the gut microbiota.

## Methods

### Patient cohort

A total of 383 patients (n=567 samples) with CD were enrolled at the University of Rennes (France) referral centre over a five-year period and provided informed consent for this observational, non-interventional study. This prospective observational study of CD patients was conducted from the standard follow-up of 383 CD patients from 2018 to 2022 including 255 patients with 1 sample, 88 patients with 2 consecutive samples, 28 patients with 3 consecutive samples, 9 patients with 4 consecutive samples, 2 patients with 5 consecutive samples and 1 patient with 6 consecutive samples, for a total of 383 patients with at least one sample (567 samples). Patients were informed of their enrolment in a prospective research database (Rennes, approved by the Commission Nationale Informatique et Liberté (CNIL) No. 1412467). Inclusion criteria were patients aged 16-80 years with a diagnosis of CD based on standard endoscopic, histological or radiological criteria. Exclusion criteria included patients with ulcerative colitis and/or ostomy. Information on sex, age, smoking, gastrointestinal surgery, Montreal classification, faecal calprotectin (FC) levels, and treatments (anti-TNF-α [infliximab, adalimumab, golimumab]; anti-integrin α-4β7 [vedolizumab]; anti-IL-12 and IL-23 [ustekinumab]; thiopurines; methotrexate [MTX]) was collected on the same day as the faecal samples, as detailed in **Table 1**. At each visit, the severity of the patient’s CD was assessed using the Harvey-Bradshaw Index (HBI) and a faecal sample was collected. HBI thresholds were used to classify patients into three groups: “remission” (CR) (HBI < 5 with no abdominal pain and no complications); “mild-moderate” (CM) (HBI = 5-8 with mild or moderate abdominal pain and no complications); and “severe” (CS) (HBI > 8 with severe abdominal pain and at least one complication) (Vermeire et al., 2010). The clinical and biological characteristics between CR, CM and CS are compared in **Table 1**. Chi2 or Kruskall-Wallis tests were performed. A P value <0.05 was considered significant.

**Table 1:** Cohort characteristics and univariate analysis of disease activity (remission/mild moderate/severe), n (%) (n= 567 samples/383 patients).

Variables	Remission (n= 291 samples)	Mild-moderate (n=177 samples)	Severe (n=99 samples)	P
Age (years)	40.09 ± 15.31 [14.37-83.38]	38.55 ± 13.94 [15.16 – 82.26]	43.55 ± 15.05 [15.8 – 77.37]	NS
Female Sex	155 (53.3%)	97 (54.8%)	64 (64.6%)	NS
Smoking	50 (17.2%)	43 (24.3%)	27 (27.3%)	NS
Gastrointestinal surgery	140 (48.1%)	75 (42.3%)	50 (50.5%)	NS
Montreal A	A1: 72 (24.7%); A2: 183 (62.9%); A3: 34 (11.7%)	A1: 29 (16.4%); A2: 122 (68.9%); A3: 25 (14.1%)	A1: 12 (12.1%); A2: 67 (67.7%); A3: 18 (18.2%)	NS
Montreal B	B1: 132 (45.4%); B2: 45 (15.5%); B3: 27 (9.3%); pB1: 62 (21.3%); pB2: 11 (3.8%); pB3: 8 (2.7%)	B1: 80 (45.2%); B2: 33 (18.6%); B3: 17 (9.6%); pB1: 33 (18.6%); pB2: 4 (2.3%); pB3: 4 (2.3%)	B1: 36 (36.4%); B2: 21 (21.2%); B3: 10 (10.1%); pB1:22 (22.2%); pB2: 6 (6%); pB3: 1 (1%)	NS
Montreal L	L1: 95(32.6%); L2:65 (22.3%); L3: 129 (44.3%); L4: 32 (11%)	L1: 57 (32.2%); L2: 30 (16.9%); L3: 88 (49.7%); L4: 29 (16.4%)	L1: 28 (28.3%); L2: 23 (23.2%); L3: 46 (46.5%); L4: 10 (10.1%)	NS
Faecal calprotectin (µg/g)	<50: 139 (47.8%); 50-250: 89 (30.6%); >250: 34 (11.7%)	<50: 49 (27.6%); 50-250: 50 (28.2%); >250: 61 (34.4%)	<50: 23 (23.2%); 50-250: 21 (21.2%); >250: 35 (35.4%)	<0.001
Anti-TNF α (%); [infliximab (%), adalimumab (%), golimumab (%)]	Anti-TNF α: 141 (%) [infliximab: 27 (9.3%), adalimumab: 117 (40.2%), golimumab: 2 (0.7%)]	Anti-TNF α: 95 (53.7%) [infliximab: 16 (9%), adalimumab: 79 (44.6%), golimumab: 2 (1.1%)]	Anti-TNF α: 49 (49.5%) [infliximab: 6 (6%), adalimumab: 42 (42.4%), golimumab: 2 (2%)]	NS
Anti-integrine α4β7: Vedolizumab (%)	5 (1.7%)	5 (2.8%)	2 (2%)	NS
Anti-interleukin (IL)-12 and IL23: Ustekinumab (%)	47 (16.2%)	38 (21.5%)	24 (24.2%)	NS
Thiopurines (%)	50 (17.1%)	36 (20.3%)	23 (23.2%)	NS
Methotrexate (MTX) (%)	23 (7.9%)	26 (14.7%)	20 (20.2%)	0.003

Montreal A (age at diagnosis): A1<16 years, A2: 17-40 years and A3: >40 years. Montreal L (Disease location): L1 ileum; L2 colon; L3 ileum-colon; L4 isolated upper disease. Montreal B (disease behaviour): B1 inflammatory; B2 structuring; B3 penetrating; p perianal disease modifier. Anti-TNFα: infliximab, adalimumab, golimumab. Anti-integrine α4β7: Vedolizumab. Anti-interleukin (IL)-12 and IL23: Ustekinumab. Thiopurines: azathioprine, 6-mercaptopurine. (CR: patients in remission; CM: patients with mild to moderate disease; CS: patients with se-vere disease). Data are (%) of samples unless otherwise stated. NS, not significant.

### Analysis of 16S rRNA gene amplicon sequencing

We used a standard protocol for 16S rRNA gene-based profiling of the faecal microbiota. In accordance with the International Human Microbiome Standards (IHMS), faecal samples were collected in a sterile container (supplied by VWR) immediately after defecation. Samples were stored at 4°C for up to 24 hours and then at - 80°C in the laboratory until DNA extraction. As described in our previous article (Buffet-Bataillon et al., 2022), DNA extraction from faecal samples was performed using MagAttract Microbial DNA (Qiagen^®^) according to the manufacturer’s instructions. Primers were designed to target the V3-V4 regions of bacteria, (PCR_341 F: 5’-CCTACGGGNGGCWGCAG-3’); (PCR_785R: 5’-GACTACHVGGGTATCTAATCC-3’). PCR products were then sequenced on the Illumina MiSeq platform following the protocol in the Illumina 16S Sample Preparation Guide. FROGS v4.0.1 was used for bioinformatic analysis of the data, following the author’s guidelines. First, reads were merged using pear (Zhang et al., 2014), primers were removed using cutadapt (Martin, 2011) and sequences shorter than 380, longer than 500 and containing N were removed. After dereplication, 218,947,691 of the 243,133,427 sequences remained. Sequences were then clustered using swarm with an aggregation distance of 1 and using the fastidious option (Mahé et al., 2015). Vsearch was used to remove chimera (12.2% of the clusters) (Rognes et al., 2016). The 500 most abundant (out of 40,014,154) clusters were retained, corresponding to 127,532,487 sequences (66.3% of 192,259,330). These Amplicon Sequence Variants (ASVs) were aligned using Blast with blast against Silva v138.1 filtered on Pintail 100 (Altschul et al., 1990; Quast et al., 2013). All reads were assigned to the lowest possible taxonomic level (species or genus) using FROGS (Escudié et al., 2018). An ASV table was then generated from the 16S rRNA gene sequencing data.

### Analysis of Amplicon Sequence Variants

As described in another previous article (Buffet-Bataillon et al., 2021), descriptive statistics and visualisation of microbiome data were performed in R (version 4.3.3) within Jupyter Notebooks (Visual Studio Code, version 1.88.0) using several packages (McMurdie and Holmes, 2013). Alpha diversity was assessed using the Chao1 and Shannon indices. The Bray-Curtis dissimilarity matrix was used to assess beta diversity, and the results were visualised using Principal Coordinates Analysis (PCoA) based on a variant of the PERMANOVA procedure (using the “adonis” function in the R “vegan” package). The Kruskal-Wallis test was used to compare the relative abundance of bacterial microbiota at the phylum and genus level in remission, mild-moderate and severe patients. Differential abundance analysis of bacterial genera between remission and severe patients was performed using DESeq2 (adjusted p < 0.05). To analyse interactions and differences between ASV in remission and severe patients, Pearson correlations were calculated and a correlation matrix and interaction network were generated using Cytoscape (version 3.10.2) (https://cytoscape.org/). The False Discovery Rate (FDR) method was used to adjust p-values for multiple testing.

### Analysis of metagenome function

PICRUSt2 is a widely used prediction tool that uses a hidden state prediction algorithm to infer function from 16S rRNA gene phylotypes (Douglas et al., 2020). Functional abundance tables were generated using PICRUSt2 (version 2.4.1) integrated into FROGS from the ASV abundance table and representative ASV sequences generated. We generated abundance tables from the databases: Kyoto Encyclopedia of Genes and Genomes (KEGG) orthologs (KO) and Enzyme Commission (EC) databases. First, we used multivariate association with linear models (MaAsLin) to find associations between CR, CM and CS and the functional abundance of KOs (Mallick et al., 2021). An FDR < 0.25 was the default setting for MaAsLin2. KO is a classification system developed by the KEGG database (Kanehisa et al., 2023). It uses a hierarchical structure to classify enzymes based on the reactions they catalyse. To better understand the role of pathways in CR and CS and to classify the pathways, the KO abundance table can be converted to KEGG pathway abundance (https://www.genome.jp/kegg/pathway.html). We then used KEGG Pathway to generate a KEGG pathway abundance table. The relative abundance of KEGG pathways and KOs between CR and CS was analysed using a two-tailed unpaired Student’s t-test. P<0.05 was considered to indicate a significant difference. In addition, we performed differential abundance analysis on the PICRUSt2 predicted abundances using DESeq2. Analogous to the binary comparisons for the microbial selection, the functional abundance table was split according to CR and CS. From the DESeq2 output, we considered as ‘significantly differentially abundant’ those functions with an estimated ‘effect’ of ≥2 (absolute value of log2 fold change or base mean).

## Results

### Changes in gut microbiota diversity, and composition with disease severity

**Figure 1:**
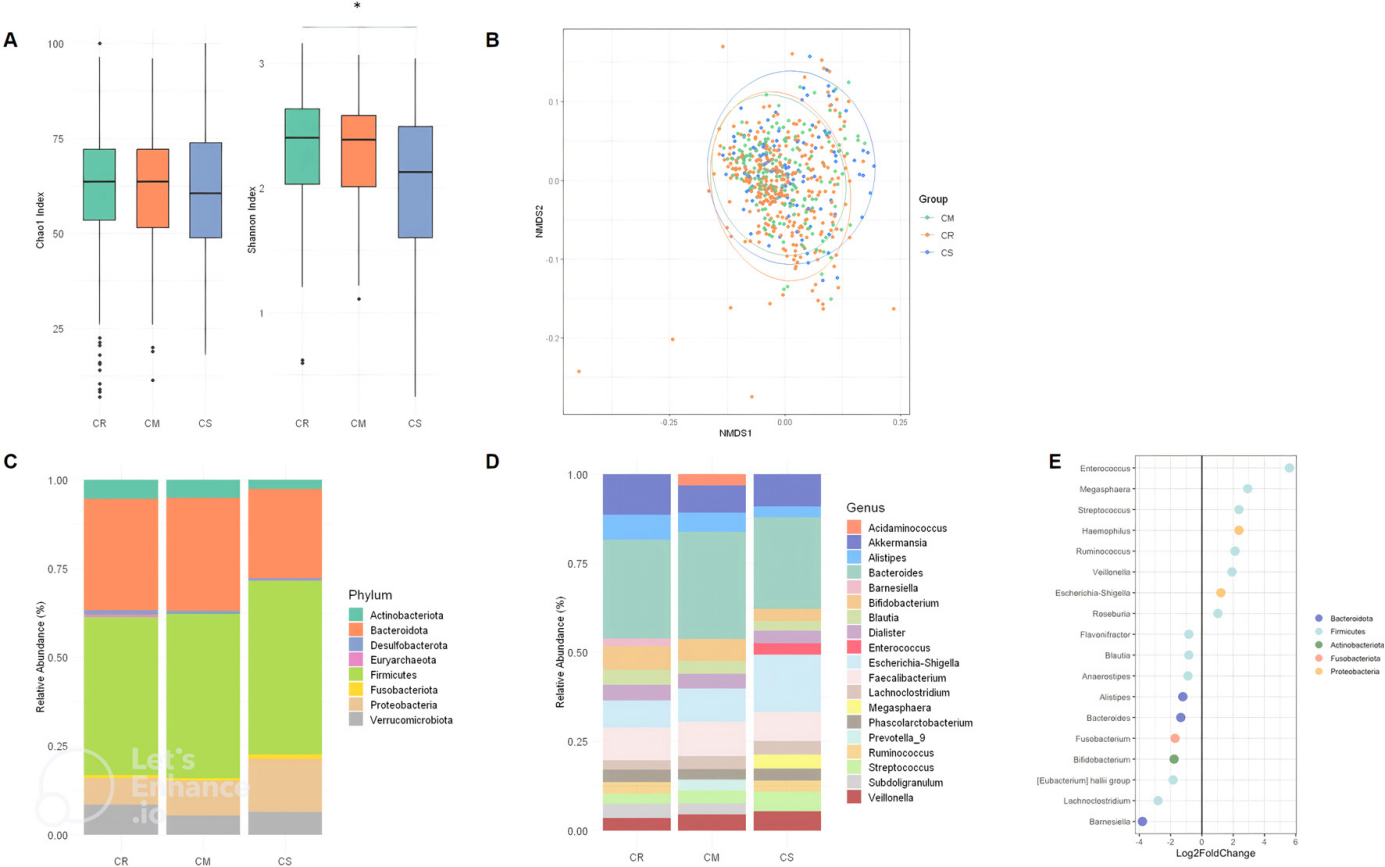
Alpha diversity index (Chao1 index and Shannon) box plot in CR, CM, CS **(A)**. Beta diversity (Bray Curtis) between CR, CM and CS. **(B)** Global composition of bacterial microbiota at the phyla level **(C)** and at the genus level **(D)** in CR, CM and CR, **(E)** DESeq2 analysis of bacterial microbiota between CR and CS: Significant (p < 0.05) log-fold changes in the abundance of bacterial genera in CS com-pared to CR. A positive log-fold change indicates an increase in abundance in CS compared to CR, whereas a negative log-fold change indicates a decrease in abundance. (CR: patients in remission; CM: patients with mild to moderate disease; CS: patients with severe disease). The symbol * means *p<0.05.

We first wanted to confirm how the composition and diversity of the gut microbiota changed in CD patients with different disease severity. A total of 567 samples from 383 different patients were included. Of these, 291 (51.3%) samples were related to remission status (CR), 177 (31.2%) samples to mild to moderate status (CM) and 99 (17, 5%) samples to severe status (CS) according to the Harvey Bradshaw Index (HBI). Clinical characteristics were comparable between CR, CM and CS (**Table 1**). As expected, significant changes in microbiota alpha and beta diversity and composition were observed between CR, CM and CS. CS had a significantly lower (p<0.01) Shannon diversity than CR, whereas the Shannon diversity of CM was similar to that of CR (**Figure 1A**). The Chao1 richness of CS was similar to that of CR or CM (p>0.05). Globally, the beta diversity of the gut microbiota differed significantly between CR, CM and CS (Permanova; p<0.05) (**Figure 1B**). At the phylum level, the abundance of Proteobacteria was higher in both CM and CS than in CR (P<0.01), whereas that of Actinobacteriota was lower (P<0.01) particularly in CS than in CR (P<0.01) (**Figure 1C**). No difference in the abundance of Firmicutes was observed between CR, CM and CS (P>0.05). At the genus level, the relative abundance of pro-inflammatory genera such as *Escherichia-Shigella*, *Veillonella*, *Megasphaera*, *Streptococcus* and *Enterococcus* increased in CS compared to CR (P<0.01), whereas anti-inflammatory genera such as *Bifidobacterium*, *Akkermansia Faecalibacterium*, *Blautia*, *Alistipes* decreased in CS compared to CR (P<0.01) (**Figure 1D**). Differential abundance analysis (DESeq2) showed the same changes for proinflammatory genera in CS compared to CR (**Figure 1E**).

**Figure 2:**
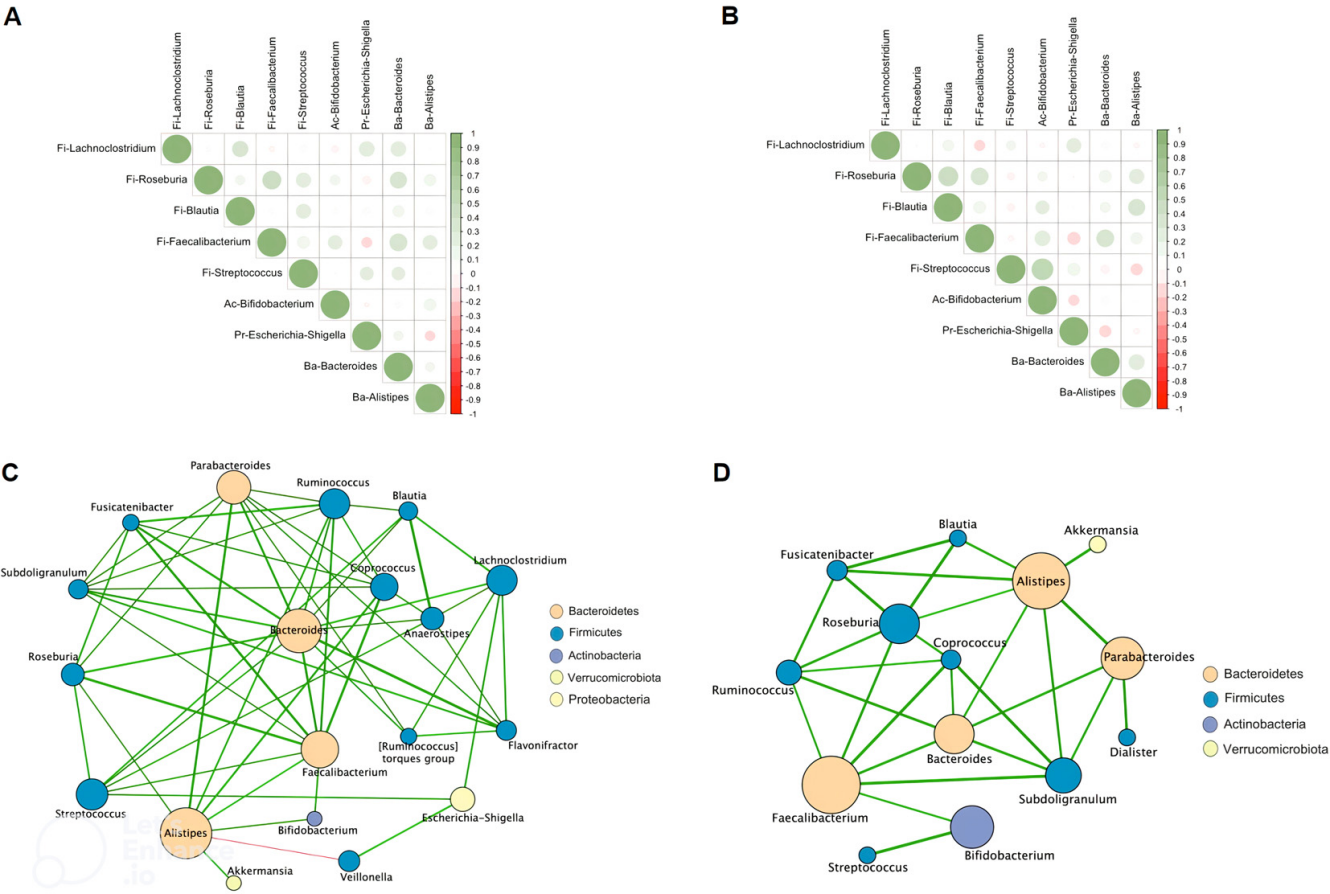
Spearman correlation matrix between bacterial genera in CR **(A)** and in CS **(B)**. Positive values (green rounds) indicate positive correlations, and negative values (red rounds) indicate inverse correlations. The shading of the round indicates the strength of the association; darker rounds are more strongly associated than lighter rounds. Molecular correlation analysis (Cytoscape software, version 3.10.2). Whole bacterial genus correlation analysis showing molecular interactions/grouping; **(C)** extracted analysis showing bacterial genus highly correlated in CR; **(D)** extracted analysis showing bacterial genus highly correlated in CS; Nodes are labelled with the weight of the bacterial genus they represent. The width of the edges connecting the nodes is proportional to the strength of the interactions. (CR: patients in remission; CS: patients with severe disease).

The striking differences and interactions between the bacterial communities were investigated using a correlation matrix and interaction network with Cystoscape (version 3.10.2) (**Figure 2**). In CR, *Faecalibacterium* was positively correlated with *Streptococcus*, *Bifidobacterium*, *Bacteroides*, *Alistipes*, and negatively correlated with *Escherichia-Shigella* (**Figure 2A**). In CS, *Faecalibacterium* remained positively correlated with *Bifidobacterium*, *Bacteroides*, *Alistipes*, but more negatively correlated with proinflammatory bacteria such as *Streptococcus* and *Escherichia-Shigella* (**Figure 2B**). The interaction network was characterised by a more restricted structure in CS compared to CR (**Figures 2C, D**). In CR, the interaction networks highlight the dense and complex genus interactions between genera supporting remission status. *Escherichia-Shigella* was associated with pro-inflammatory bacteria such as *Veillonella*, *Streptococcus*, but also with anti-inflammatory bacteria such as *Lachnoclostridium* (**Figure 2C**). In CS, the anti-inflammatory bacteria *Akkermansia* was only associated with *Alistipes*, which in turn had limited correlations with *Blautia*, *Subdoligranulum*, *Bacteroides*, *Roseburia*, *Fusicatenibacter*, and *Bifidobacterium*.(**Figure 2D**).

### Changes in functional pathways across severity status

**Figure 3:**
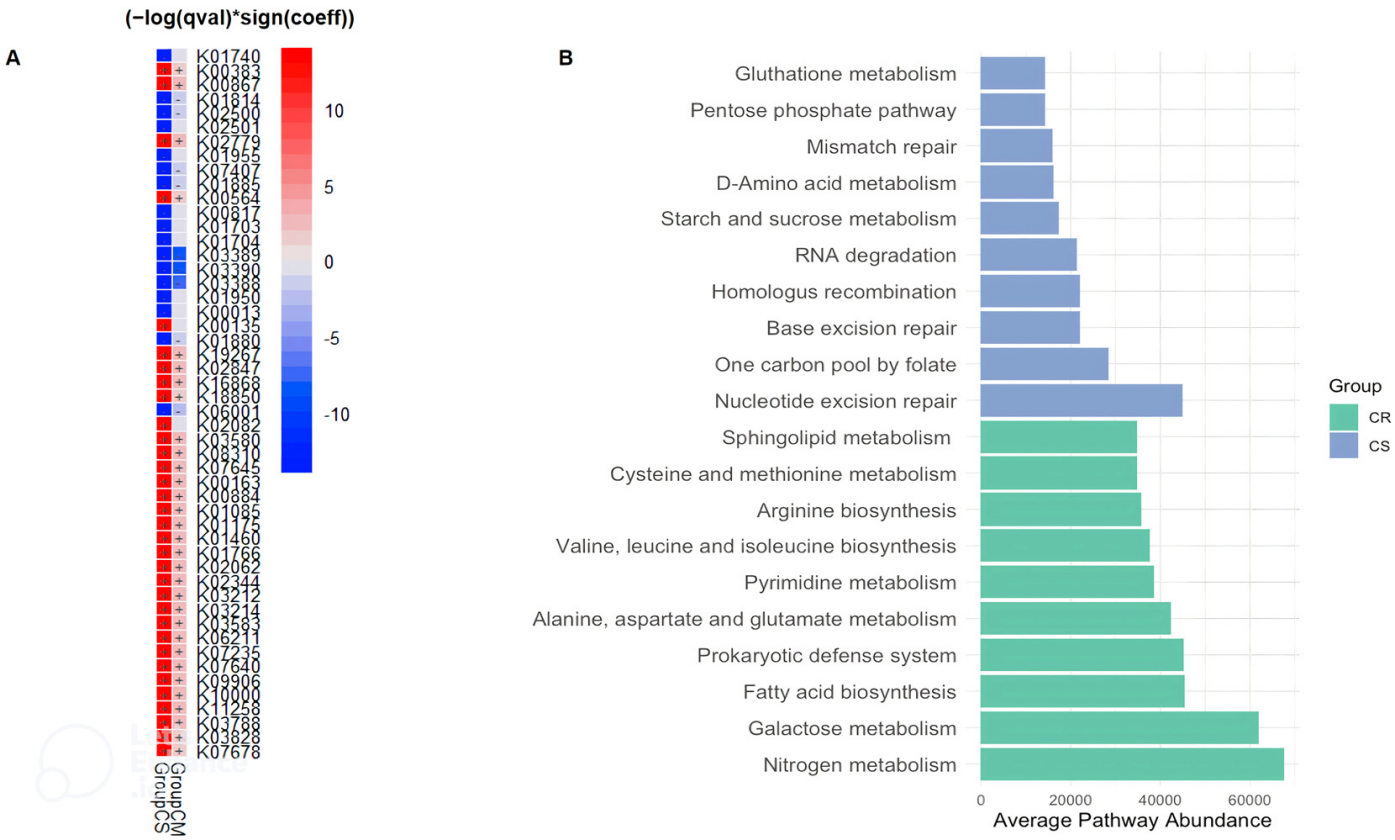
Distinct KEGG Orthologs (KO) **(A)** and KEGG Pathway Variations **(B)** in CD Patients Related to Severity Status. **(A)** According to the MaAsLin2 regression coefficient, there was a gradation of overexpression or underexpression of KEGG orthologs (KO) from CR, then CM and finally CS. **(A)** The heatmap shows the comparative analysis for functions between severity of CD patients. Red and blue in the heatmap represent functions enriched and depleted between CS or CM and CR (FDR < 0.05, MaAsLin2), respectively. **(B)** Microbial KEGG pathways with significantly altered abundances in CR and CS **(B)** (Green: CR; Blue: CS). Basic metabolism (most amino acid and fatty acid biosynthesis) was decreased in abundance in CS, whereas metabolism of biological antioxidants (glutathione metabolism; pentose phosphate pathway) and numerous DNA, RNA repair pathways beneficial for oxidative stress were increased in CS. (CR: patients in remission; CM: patients with mild to moderate disease; CS: patients with severe disease).

Taken together, the alpha and beta diversity, composition, and network data confirm that the gut microbiota changes between CR, CM and CS. We next wanted to assess how these observed changes affect the functionality of the gut microbiota. To explore the functional potential of the gut microbiota and its role in gut inflammation, we used PICRUSt 2 which refers to gene families such as KEGG orthologs (KOs) and Enzyme Classification numbers (ECs). First, we used multivariate association with linear models (MaAsLin2) to find associations between the KO abundance table and CR, CM and CS (**Figure 3A**). Then, to better understand the role of pathways in CR and CS groups and to classify pathways, the KO abundance table was converted to KEGG pathway abundance.

**Figure 4:**
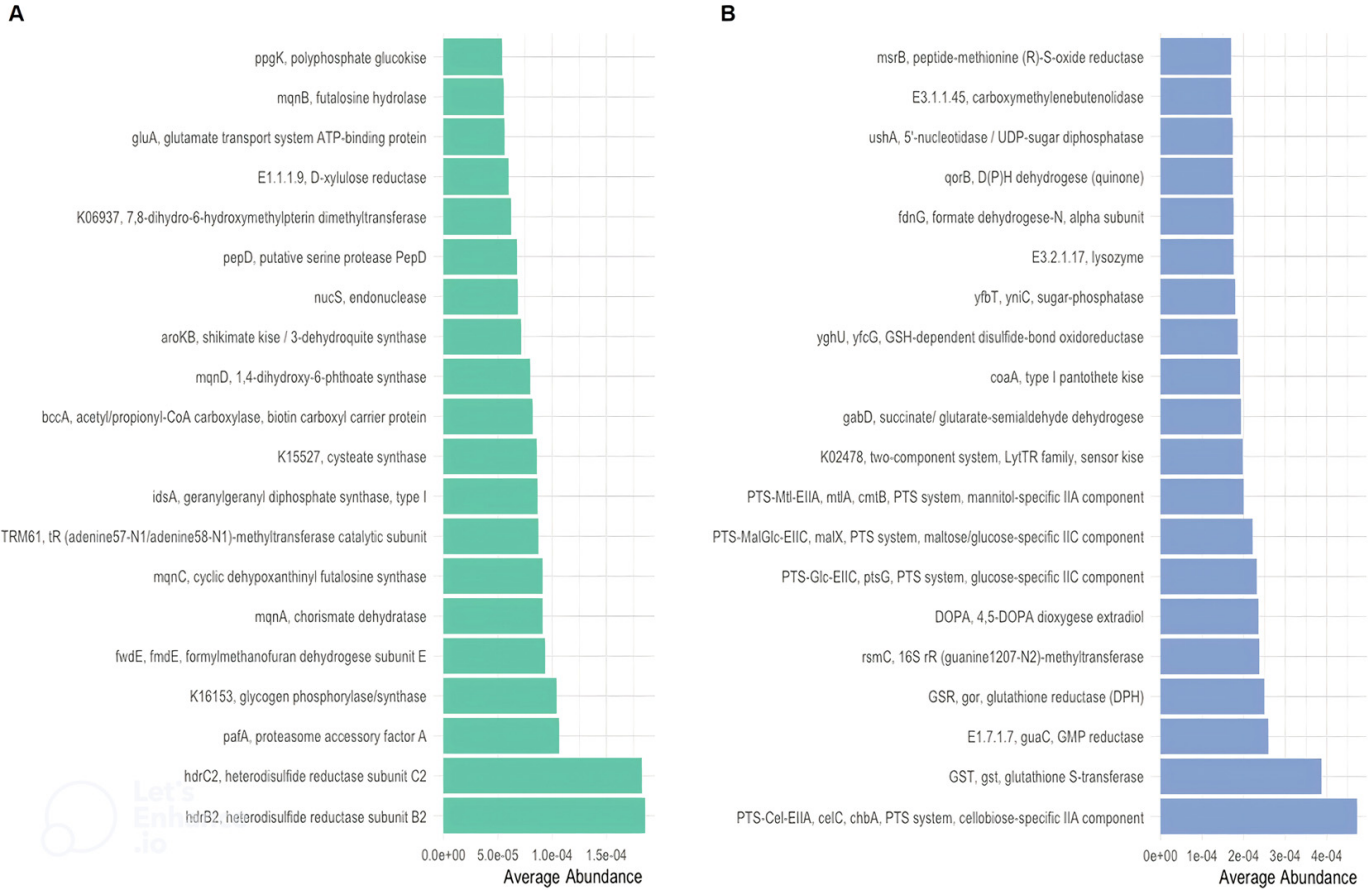
Distinct KEGG enzyme (EC) in CR and in CS. Distinct KEGG enzyme (EC) were significantly altered in abundance in CR **(A)** and CS **(B)**. KEGG enzymes (EC) were analysed for significant association with severity status. Metabolites related to oxidative stress (glutathione (GST;GSR;GSH) and regulatory systems (TCS)) and to optimal nutrient utilisation (carbohydrate transport (PTS)) were upregulated, whereas basic biosynthetic processes were downregulated. (CR: patients in remission; CS: patients with severe disease).

**Figure 5:**
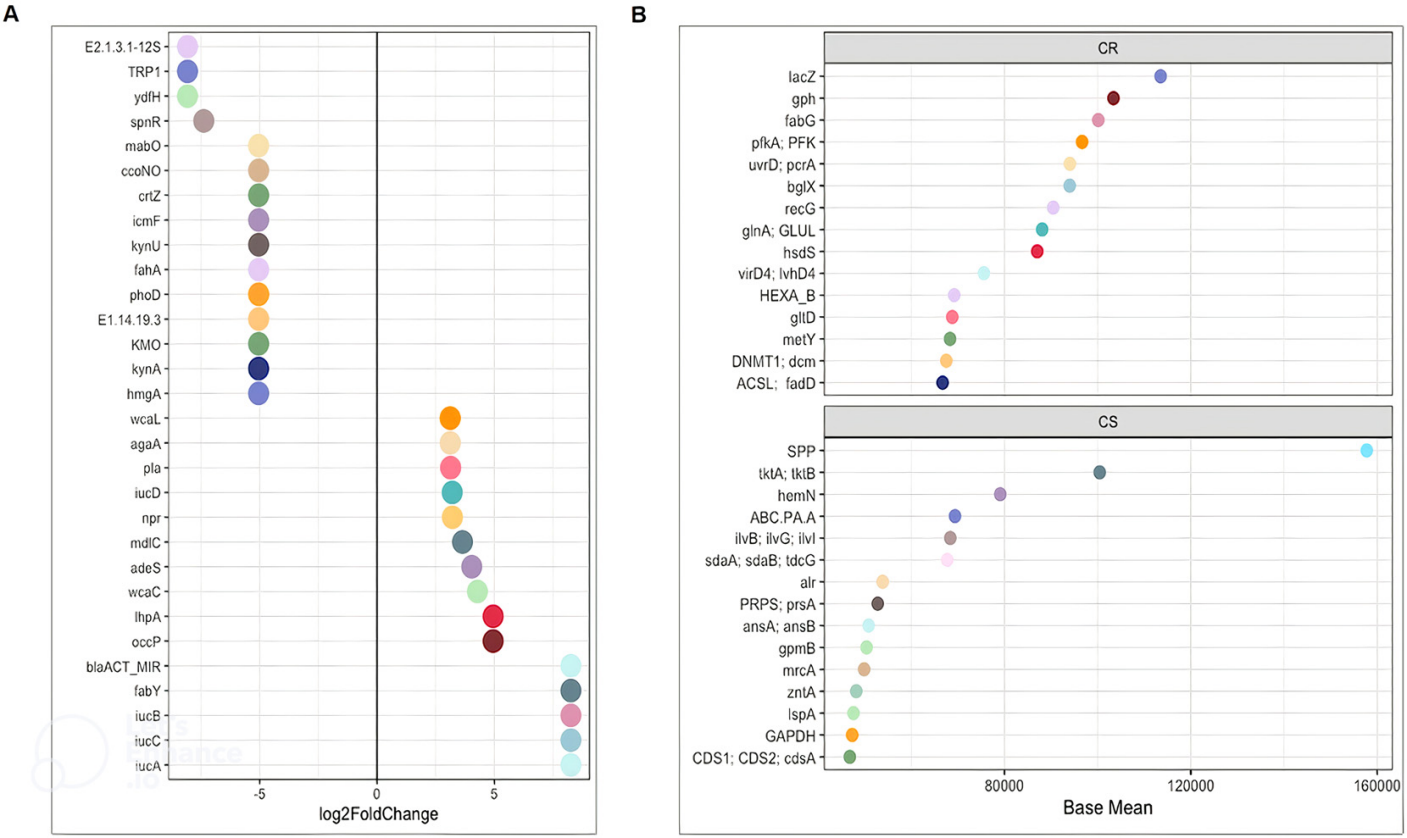
DESeq2 analysis of functions between CR and CS **(A)** Log2FoldChange and **(B)** Base Mean. The significantly differentially enriched and depleted functions between CR and CS (adjust p < 0.05) with their log2 fold changes (in the x-axis) or with their base mean were visualised by scatterplots. Functions were plotted on the left axis of the scatterplots. Each point on a scatterplot represents one bacterial function. (CR: patients in remission; CS: patients with severe disease).

The average pathway abundance (**Figure 3B**) and KO abundance (**Figures 4A, B**) in CR and CS were analysed. In addition, we performed a differential abundance analysis between CR and CS on KO abundance abundances using DESeq2 with an estimated “effect” of ≥2 (Log2 fold change (**Figure 5A**) or base mean (**Figure 5B**).

MaAsLin2 showed that the changes in the gut microbial functional pathways were graded from CR to CM, and then from CM to CS. There was a gradient of over- and under-expression of KOs from CR to CM and from CM to CS (**Figure 3A**). Among the 50 KOs differentially expressed between groups, 31 KOs and 9 KOs were over-expressed and under-expressed in CS compared to CR, respectively, and intermediate expressed in CM. These results revealed distinct metabolic signatures between the CR and CS groups, highlighting potential molecular mechanisms underlying CD pathogenesis. Therefore, we decided to focus our analysis with different statistical approaches of functional microbiota in CS *versus* CR.

### Amino acid biosynthesis and carbohydrate metabolism are reduced in CS whereas amino acid and carbohydrate transport are increased

Considering only the contrast between CS and CR, a total of 1030 and 459 different KOs were differentially abundant (p <0.05). The average abundance of almost all amino acid metabolic pathways such as alanine, arginine, aspartate, cysteine, glutamate, isoleucine, leucine, methionine, valine was decreased in CS (**Figure 3B**). Focusing on KOs involved in tryptophan metabolism, the abundance of several KOs was decreased in CS: the proteasome accessory factor A (pafA), shikimate kinase (aroKB) (**Figure 4A**) and several KOs were underexpressed as K06001 trpB, tryptophan synthase beta chain [EC:4.2.1.20]; (**Figure 3A**); as kynA, tryptophan 2,3-dioxygenase [EC:1.13.11.11], KMO [EC:1.14.13.9], KynU [EC:3.7.1.3], TRP (**Figure 5A**). The amino acid transport system with K02062 thiQ, thiamine transport system ATP-binding protein; K10000 artP, arginine transport system ATP-binding protein [EC:3.6.3.-] (**Figure 3A**) and ABC.PA.A, polar amino acid transport system ATP-binding protein [EC:3.6.3.21] was overexpressed in CS (**Figure 5B**).

We found a decrease in carbohydrate metabolism in CS, including D-xylulose reductase (EC: E1.1.1.9), glycogen phosphorylase/synthase (K16153), polyphosphate glucokinase (ppgK) and acetyl-CoA/propionyl-CoA carboxylase (bccA) (**Figure 4A**) or E2.1.3.1-12S and spnR (**Figure 5A**), whereas increased KO was associated with carbohydrate transport as specific PTS transport systems for cellulose (PTS-Cel-EIIA celC chbA), mannitol (PTS-Mtl-EIIA mtlA cmtB), maltose/glucose (PTS-MalGlc-EIIC malX), and glucose (PTS-Glc-EIIB ptsG); K02779 PTS-Glc-EIIC, ptsG, PTS system, glucose-specific IIC component (**Figure 3A**) were strongly present. CS showed a decrease in lipid metabolism as indicated by a reduction in fatty acid biosynthesis (**Figure 3B**).

### Extreme functional changes in CD include changes associated with oxidative stress

Oxidative stress refers to increased intracellular levels of reactive oxygen species (ROS) that cause damage to DNA, proteins and membrane lipids. We observed increases in DNA and RNA repair pathways such as nucleotide excision repair, base excision repair, and homologous recombination, RNA degradation (**Figure 3B**), peptide-methionine (R)-S-oxide reductase (msrB) (**Figure 4B**); and K02344 holD, DNA polymerase III subunit psi [EC:2.7.7.7]; K03583 recC, exodeoxyribonuclease V gamma subunit [EC:3.1.11.5]; K03580 hepA, ATP-dependent helicase HepA [EC:3.6.4.-] (**Figure 3A**) in CS. This was associated with a global decrease in pyrimidine and nucleotide biosynthetic modules in CS (**Figure 3B**), such as dihydroxy-methylpterin dimethylallyltransferase (K06937), nucS endonuclease, and tRNA (adenine57-N1/adenine58-N1)-methyltransferase (TRM61) (**Figure 4A**), mabO and phoD (**Figure 5A**) and K01950 E6.3.5.1, NADSYN1, QNS1, nadE, NAD+ synthase (glutamine hydrolysing) [EC:6.3.5.1]; K01880 GARS, glyS1, glycyl-tRNA synthetase [EC:6.1.1.14] (**Figure 3A**).

### To counteract the damaging effects of ROS, glutathione (GSH) is the most important biological antioxidant

We observed an increase in the abundance of glutathione metabolism genes in CS (**Figure 3B**), notable KOs being glutathione S-transferase (GST, gst), glutathione reductase (NADPH) (GSR, gor; [EC:1. 8.1.7]), K01460 gsp, glutathionylspermidine amidase/synthetase [EC:3.5.1.78 6.3.1.8] and GSH-dependent disulfide bond oxidoreductase (yghU, yfcG) regenerate glutathione (**Figures 4B**, **3A**). The pentose phosphate pathway pentose is required to regenerate oxidised glutathione back to its reduced form. The pentose phosphate pathway E2.2.1.1, tktA, tktB, transketolase [EC:2.2.1.1]; PRPS, prsA, ribose phosphate pyrophosphokinase [EC:2.7.6.1] were also overrepresented in CS (**Figure 5B**). In contrast, CR showed a higher abundance of genes involved in ubiquinone biosynthesis: mqnB, mqnD, mqnC and mqnA (**Figure 4A**).

### Finally, genes involved in pathogenic processes, such as regulatory systems and virulence factors were overrepresented in CS

The two-component regulatory system (TCS) is the predominant regulatory system for bacteria to sense and respond to environmental changes, and can therefore be considered an essential requirement for their pathogenicity. TCS was significantly present in CS as a two-component LytTR sensor kinase system (K02478) (**Figure 4B**). Bacteria use quorum sensing to regulate a variety of functions, including virulence and biofilm formation. Quorum sensing detection used by enterobacteria was significantly upregulated in CS as: K07640 cpxA, two-component system, OmpR family, sensor histidine kinase CpxA [EC:2.7.13.3]; K07645 qseC, two-component system, OmpR family, sensor histidine kinase QseC [EC:2.7.13.3] (**Figure 3A**). Virulence factors appeared to be predominantly related to iron uptake as KOs related to aerobactin biosynthesis iucD, iucA, iucC, and iucB, KO related to iron porphyrin metabolism (**Figure 5A**): hemN, hemZ, oxygen-independent coproporphyrinogen III oxidase [EC:1.3.98.3] were overexpressed in CS (**Figure 5B**). In addition, adherence/invasion was prominently enriched in CS as adhesin-related KOs such as pla, plasminogen activator [EC:3.4.23.48] (**Figure 5A**), as well as KOs related to biofilm formation in E.coli: K07678 barA, gacS, varS, two-component system, NarL family, sensor histidine kinase BarA [EC:2.7.13.3] (**Figure 3A**). Moreover, KOs conferring resistance to β-lactam antibiotics were also identified, such as adeS and blaACT_MIR, ddl, D-alanine-D-alanine ligase [EC:6.3.2.4]; alr, alanine racemase [EC:5.1.1.1]; mrcA, penicillin binding protein 1A [EC:2.4.1.129 3.4.16.4]. KO, mrcA, penicillin binding protein 1A [EC:2.4.1.129 3.4.16.4] (**Figure 5**).

## Discussion

We investigated microbial community functions from taxonomic profiles in CR, CM and CS.

First, our study confirms that gut microbiota dysbiosis is strongly associated with CD severity. Our results confirmed a significant decrease in Shannon’s alpha diversity index in CS and a dissimilarity between CR, CM and CS. In particular, Proteobacteria were significantly more abundant in CS than in CR and CM. At the genus level, pro-inflammatory bacteria such as *Escherichia-Shigella*, *Veillonella*, *Megasphaera*, *Streptococcus* and *Enterococcus* were identified as biomarkers. In contrast, the anti-inflammatory bacteria: *Faecalibacterium*, *Akkermansia*, *Alistipes*, *Blautia*, *Bifidobacterium*, were more abundant in CR. These results were consistent with previous reports in the literature (Sokol et al., 2008; Joossens et al., 2011; Gevers et al., 2014; Tedjo et al., 2016a; Pascal et al., 2017a; Zuo and Ng, 2018; Franzosa et al., 2019; Lloyd-Price et al., 2019; Caparrós et al., 2021; Deleu et al., 2021a). In addition to the general analysis of the differential abundance of phyla and genera in patients in remission or in severity status, our study defined distinct networks of taxa associations that are essential for mechanically capturing the structure and maintenance of the microbial community. According to Yilmaz et al, *Faecalibacterium* was positively correlated with anti-inflammatory bacteria such as *Bifidobacterium* and negatively correlated with pro-inflammatory genera such as *Escherichia-Shigella* (Yilmaz et al., 2019). Furthermore, our results support that the robustness of microbial networks and their interdependent structure are associated with CR microbiota, whereas a loose structure and an increase in *Enterobacteria* characterise CS microbiota (Mondot et al., 2016).

**Figure 6:**
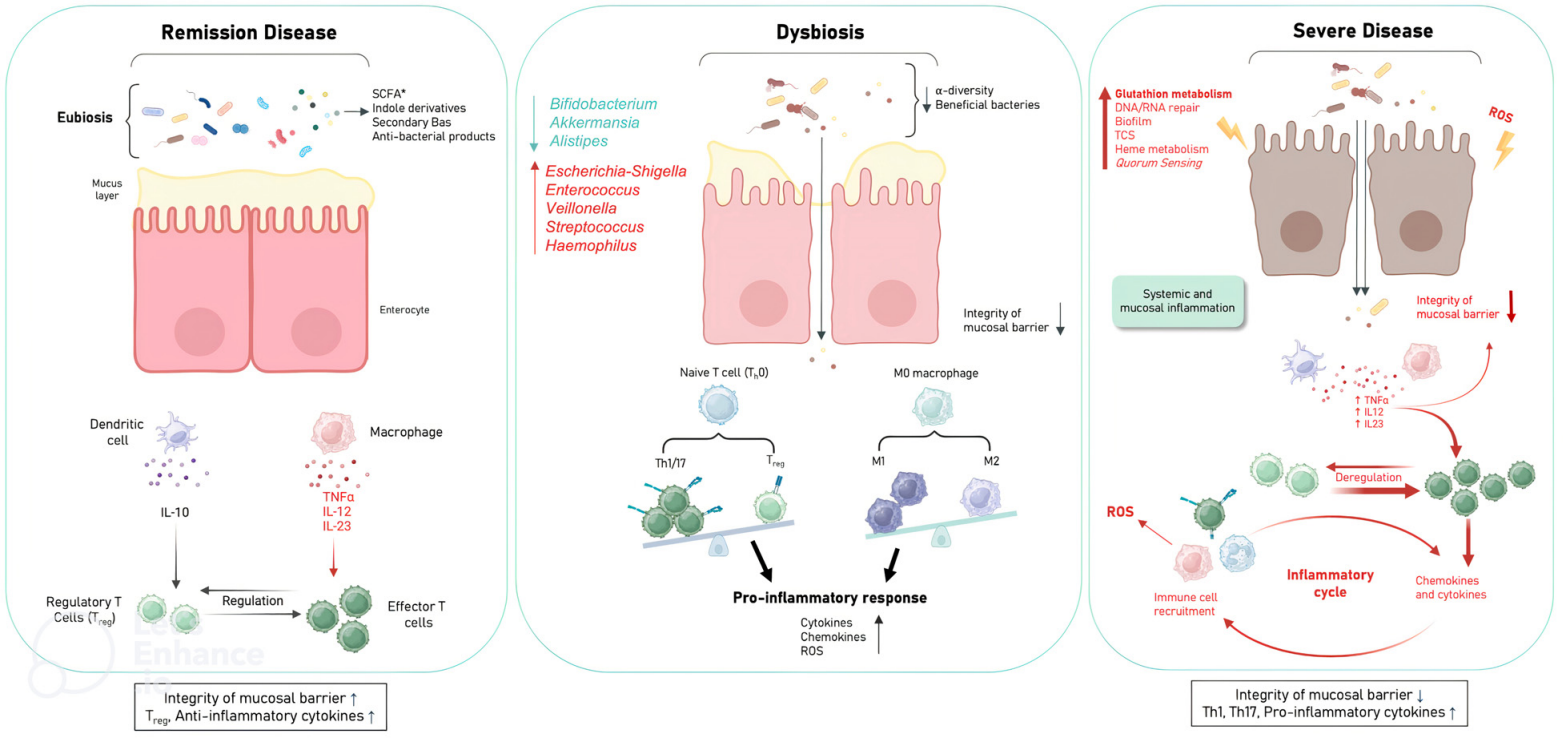
Schematic of the mechanism hypothesis of gut microbiota dysfunction in relation to CD severity. Disease in remission is characterised by integrity of the mucosal barrier; presence of Treg; anti-inflammatory cytokines due to eubiosis and microbial pathways involving amino acid biosynthesis (glutamate (EGFR activation), tryptophan (indole derivatives: AhR activation), carbohydrate biosynthesis (SCFAs (butyrate, acetate, propionate): GPR activation), lipid biosynthesis (secondary BAs: TGR5 and FXR activation). ROS production by immune cells is also prevented by ubiquinone biosynthesis. Mild to moderate disease is characterised by the establishment of dysbiosis with the loss of beneficial anti-inflammatory bacteria and the presence of pro-inflammatory bacteria, thus decreasing the basal beneficial metabolism to down regulate inflammation leading to mucosal barrier damage, over ROS production, pro-inflammatory responses pro-inflammatory cytokines. Severe disease is characterised by loss of mucosal barrier integrity; presence of Th1, Th17; pro-inflammatory cytokines with excessive amounts of ROS (DNA, RNA repair; glutathion metabolism), microbial virulence factors to invade the host (TCS, quorum sensing, heme metabolism) and to evade host defences (biofilms). (Treg, Regulatory T cells; EGFR, epidermal growth factor receptor; AhR, aryl hydrocarbon receptor; SCFA, short chain fatty acids (Butyrate, acetate, propionate); GPRs, G protein-coupled receptors; Ba, bile acids; FXR, farnesoid X receptor; TGR5, G protein-coupled bile acid receptor 1; TCS, two-component systems; ROS, Reactive oxygen species).

Second, the functional analysis of the gut microbiota provides new insights into the relationship between dysbiosis and CD, with possible causality, opening the way to new therapeutic approaches (**Figure 6**). By combining shifts in the abundance and expression of functional modules, two major imbalances in the progression of CD severity were identified:

- Depletion of fundamental microbial pathways associated with amino acid, carbohydrate and lipid biosynthesis, while increasing amino acid and carbohydrate transport in CS.
- Involvement of oxidative stress and underlying biological mechanisms such as DNA, RNA repair, glutathione, siderophore, biofilm formation, suggesting a potentially invasive “pathobiont” in CS.

### Amino acid and carbohydrate biosynthesis were reduced in CS, suggesting that their metabolites may be drivers of an altered gut immune system

Notably, the depletion of microbial pathways associated with the glutamate metabolism was associated with CD severity status. Indeed, several studies have highlighted the critical role of glutamate in maintaining mucosal integrity (DeMarco et al., 2003; De-Souza and Greene, 2005), possibly by preventing disruption of tight junctions via transactivation of the epidermal growth factor receptor, which mediates intestinal epithelial cell proliferation (Basuroy et al., 2005).

In recent years, an increasing number of studies have shown that disorders of tryptophan metabolism are strongly associated with CD (Ding et al., 2020; Lavelle and Sokol, 2020). Gut microbiota dysbiosis induces tryptophan metabolite alterations leading to CD progression, which is mainly based on reduced indole derivatives and AhR activity (Monteleone et al., 2011; Nikolaus et al., 2017). The gut microbiota is the primary source of endogenous AhR ligands. Consistent with other studies, the reduction in tryptophan metabolism in our study was associated with the reduction in *Bifidobacterium* (Agus et al., 2018; Roager and Licht, 2018).

We found a decrease in carbohydrate metabolism in CS, suggesting a possible decrease in SCFA production by the gut microbiota. SCFAs (butyrate, acetate, propionate) act as signalling molecules via G-protein coupled receptors (GPRs) in various types of host cells (De Preter et al., 2013; Makki et al., 2018; Parada Venegas et al., 2019; Lloyd-Price et al., 2019). Mucin levels in goblet cells, antimicrobial peptides in Paneth cells, and tight junction proteins in intestinal epithelial cells are upregulated by GPR activation. In addition, the secretion of pro-inflammatory cytokines (TNF-alpha, IL-2, IL-6, IL-12, IL-23) by macrophages, the expression of den-dritic cell-migrated proteins (CXCL, CD40) and HDAC activity are inhibited by SCFAs.

Treg differentiation and their secretion of anti-inflammatory cytokines such as IL-10 are in-duced by histone 3 acetylation, which is enabled by HDAC inhibition. Similarly, IL-10 secretion by dendritic cells is promoted by SCFAs. Finally, IgA production by B cells is induced by SCFAs (Campbell et al., 2023). With accumulating data suggesting regulatory functions of SCFAs in a wide range of immune cells, they represent a new frontier in the treatment of intestinal inflammation in animal models and eventually in CD patients (Sun et al., 2017).

Bile acids (BAs) are synthesised from lipids as cholesterol in hepatocytes and secreted into the duodenum. BAs are further metabolised by the gut microbiota. Bacteroidota and *Bifidobacterium* are extensively involved in the conversion of BAs, the abundance of which has been shown to be reduced in CD patients (Duboc et al., 2013; Lavelle and Sokol, 2020). Dysbiosis affects the composition of BAs. We found a decrease in fatty acid biosynthesis and in the abundance of Bacteroidota and *Bifidobacterium* in CS, which in turn may have an impaired ability to deconjugate and convert BAs into conjugated secondary BAs. Secondary BAs act as high-affinity ligands for TGR5 and FXR, the activation of which exerts immunomodulatory and anti-inflammatory effects (Duboc et al., 2013; Hang et al., 2019; Campbell et al., 2020; Fiorucci et al., 2021). Thus, gut dysbiosis and subsequent abnormal BA profile could exacerbate CD progression by inhibiting TGR5 and FXR activity.

In summary, we can hypothesise that the decrease in basic microbial pathways such as tryptophan and carbohydrate metabolism led to a lower amount of bioactive metabolites required to activate the AhR and GPRs, respectively, in order to down-regulate the gut inflammation. Due to dysbiosis, BA metabolism could be altered and participate in the chronic inflammatory loop of CD. On the contrary, in CS we found an increase in carbohydrate and amino acid transport. The characteristic of these specific transport systems is that they provide microbiota-integrated systems that ensure optimal utilisation of carbohydrates and amino acids in stressful situations (Kotrba et al., 2001; Kandasamy et al., 2018).

### Significant functional changes in CD include alterations in oxidative stress metabolism

Oxidative stress results from an imbalance between the production of ROS and the defence system responsible for their detoxification. Persistent ROS in the gut environment play a key role in chronic inflammation, immune responses and DNA damage (Wang et al., 2022). ROS are primarily produced by cells of the immune system, mainly macrophages, dendritic cells, B lymphocytes and polymorphonuclear neutrophils. Excessive amounts of ROS, which directly cause various forms of DNA damage, including single-strand breaks, double-strand breaks and DNA base sequence changes, can also be generated by the dysbiotic microbiota (Li et al., 2021; Wang et al., 2022; Barnes et al., 2022; Ray et al., 2022). Indeed, we observed an increase in DNA and RNA repair and a decrease in DNA biosynthesis in CS. In contrast, CR showed a higher abundance of genes involved in ubiquinone biosynthesis. The physiological role of ubiquinone in bacteria is to regulate energy metabolism, gene expression and prevent oxidative stress (Abby et al., 2020). In addition, our results showed an increase in glutathione metabolism and the pentose phosphate pathway, which is necessary for the regeneration of oxidised glutathione in the CS. This reflects a mechanism by which the gut microbiota respond to inflammation-induced oxidative stress. Proteobacteria and some Streptococci and Enterococci synthesise glutathione, which helps them to grow under oxidative stress (Masip et al., 2006). Accordingly, Enterobacteria, streptococci, and enterococci were enriched in CS. In addition, the role of glutathione in virulence includes the activation of virulence gene expression and contributes to optimal biofilm formation (Ku and Gan, 2021). Thus, overproduction of ROS causes oxidative stress and microbiota-induced inflammation, which are strongly linked in CD, initiating a vicious cycle of mucosal barrier damage. This can lead to increased mucosal permeability, loss of protection, which favours intestinal invasion by inflammatory bacteria, which in turn can stimulate further inflammation and ROS production (Rapozo et al., 2017; Khanna and Raffals, 2017; Wang et al., 2022).

### Significant functional changes in CD indicate that virulence factors are required to invade the host, evade host defences and participate in the establishment of inflammation

Virulence factors highlight the importance of signalling systems in the context of host-microbiota interactions. The first signalling systems in virulence processes are two-component systems (TCS), which are signalling mechanisms in bacteria that allow intracellular changes from extracellular cues and allow bacteria to adapt very quickly to changes in environmental conditions (Stock et al., 2000). TCS allow bacteria to control a variety of processes such as metabolism, oxidative stress or pathogenicity. Not only do these systems modulate oxidative stress and metabolism, but TCS also modulate virulence traits through diverse mechanisms such as iron uptake, and biofilm formation (Shaw et al., 2022). Interestingly, the TCS that are predominant in CS compared to CR are LytTR-type histidine kinase/response regulator systems, as demonstrated in *Escherichia coli* (Jung et al., 2012). The second signalling system involved in virulence processes is quorum sensing (QS). QS is a generalised cell-to-cell communication strategy that allows bacteria to coordinate their phenotypes via chemical signalling. Phenotypes such as antibiotic resistance, and the production of virulence factors are known to be genetically regulated by QS and dependent on population density (Sharma et al., 2024). QS is thought to provide a mechanism for pathogenic bacteria to minimise host immune responses by delaying the production of tissue-damaging virulence factors until sufficient bacteria have accumulated and are ready to overwhelm host defences and cause inflammation. Interestingly, KOs associated with QS recognition used by enterobacteria were significantly present in CS.

In order to invade the host, bacterial pathogens must synthesise heme or acquire heme from the host; however, host heme is sequestered in high-affinity hemoproteins. Bacteria have developed sophisticated strategies to acquire heme from host sources or from bacteria (Choby and Skaar, 2016). Aerobactin, a siderophore produced by Escherichia coli, is one of the strategy to capture available iron from other bacteria present (Micenková et al., 2018). Remarkably, KOs related to iron uptake, such as Aerobactin biosynthesis and iron porphyrin metabolism, were predominant in CS.

To evade the host defences, biofilms are communities of cells attached to surfaces and held together by a self-produced extracellular matrix. The matrix consists of different extracellular DNA molecules, proteins and polysaccharides, depending on the bacterial species (Joo and Otto, 2012). Cells in the biofilm state show increased protection against antibiotics and the host immune response molecules (Flemming and Wingender, 2010). Other studies have shown that KOs associated with biofilm formation in Escherichia coli are overabundant and overexpressed in CS (Darfeuille-Michaud et al., 2004; Baumgart et al., 2007). This biofilm allows escape from host defence and could persist despite antibiotic therapy. In addition, KOs conferring resistance to β-lactam antibiotics have been identified in CS.

Overall, metabolic pathways in CS are characterised by a strong coherence with the lifestyle of highly auxotrophic bacteria such as the “pathobiont” Escherichia coli or related genera (Iwasaki et al., 2021).

The limitation of our study is that it is an observational, non-interventional study of 383 patients (n=567 samples), of which 128 patients had more than 2 samples (n=312 samples), but with the important number of samples, it allowed us to elucidate the potential mechanisms and pathways of gut microbiota imbalance involved in the development of CD, providing a basis for updating the treatment strategies for CD. With the subset of 128 patients from the cohort with multiple temporal faecal sampling during the longitudinal study (n=312 samples in total), we aim to further exploit the microbiota-modulating effect of immunomodulators (azathioprine, 6-mercaptopurine, methotrexate), and biologic therapies (e.g., anti-TNFα: infliximab, adalimumab, golimumab; anti-integrin α4β7: vedolizumab; anti-interleukin (IL)-12 and IL23: ustekinumab) to establish a chain of events and determine whether restoration of eubiosis and pathways precedes, follows, or coincides with remission.

In conclusion, the data presented here confirm that CS is associated with a dysbiosis characterised by changes in the Proteobacteria phyla. These changes in bacterial composition were associated with major gut microbiota dysfunction in CD, linked to impaired basal metabolism and virulence factors of the microbiota in an environment of oxidative stress.

Our results suggest that two complementary approaches to restore eubiosis in the microbiota of CD patients could be: 1) rebalancing the microbiota and 2) eliminating the oxidative stress. This should result in reversing biofilm formation and rebalancing the microbiota with the ultimate goal of protecting the intestinal mucosa and reducing relapse. Finally, it will be exciting to support the development of ‘personalised microbiota-targeted’ therapy for CD based on microbial metabolic function profiles.

## Data Availability

The datasets presented in this study can be found in online repositories. Raw reads have been deposited in the Sequence Read Archive (SRA) under the project accession number PRJNA1179364.
